# The Importance of Cursive Handwriting Over Typewriting for Learning in the Classroom: A High-Density EEG Study of 12-Year-Old Children and Young Adults

**DOI:** 10.3389/fpsyg.2020.01810

**Published:** 2020-07-28

**Authors:** Eva Ose Askvik, F. R. (Ruud) van der Weel, Audrey L. H. van der Meer

**Affiliations:** Developmental Neuroscience Laboratory, Department of Psychology, Norwegian University of Science and Technology, Trondheim, Norway

**Keywords:** high-density electroencephalography, cursive handwriting, typewriting, temporal spectral evolution, digital era, learning in the brain, educational psychology

## Abstract

To write by hand, to type, or to draw – which of these strategies is the most efficient for optimal learning in the classroom? As digital devices are increasingly replacing traditional writing by hand, it is crucial to examine the long-term implications of this practice. High-density electroencephalogram (HD EEG) was used in 12 young adults and 12, 12-year-old children to study brain electrical activity as they were writing in cursive by hand, typewriting, or drawing visually presented words that were varying in difficulty. Analyses of temporal spectral evolution (TSE, i.e., time-dependent amplitude changes) were performed on EEG data recorded with a 256-channel sensor array. For young adults, we found that when writing by hand using a digital pen on a touchscreen, brain areas in the parietal and central regions showed event-related synchronized activity in the theta range. Existing literature suggests that such oscillatory neuronal activity in these particular brain areas is important for memory and for the encoding of new information and, therefore, provides the brain with optimal conditions for learning. When drawing, we found similar activation patterns in the parietal areas, in addition to event-related desynchronization in the alpha/beta range, suggesting both similarities but also slight differences in activation patterns when drawing and writing by hand. When typewriting on a keyboard, we found event-related desynchronized activity in the theta range and, to a lesser extent, in the alpha range in parietal and central brain regions. However, as this activity was desynchronized and differed from when writing by hand and drawing, its relation to learning remains unclear. For 12-year-old children, the same activation patterns were found, but to a lesser extent. We suggest that children, from an early age, must be exposed to handwriting and drawing activities in school to establish the neuronal oscillation patterns that are beneficial for learning. We conclude that because of the benefits of sensory-motor integration due to the larger involvement of the senses as well as fine and precisely controlled hand movements when writing by hand and when drawing, it is vital to maintain both activities in a learning environment to facilitate and optimize learning.

## Introduction

Digital devices are increasingly replacing traditional writing by hand (Longcamp et al., 2006; Kiefer et al., 2015), and as both reading and writing are becoming more and more digitized at all levels of education, it is crucial to examine the long-term implications of this practice that are still largely unknown (Mangen and Balsvik, 2016; Patterson and Patterson, 2017). Despite several studies supporting the benefits for learning when taking notes by hand compared to laptop note-taking (e.g., Longcamp et al., 2005; Smoker et al., 2009; James and Engelhardt, 2012; Mueller and Oppenheimer, 2014; Van der Meer and Van der Weel, 2017), it is still unclear how computer use impacts student productivity and learning (Patterson and Patterson, 2017). Due to contradictory results, it has been hard to achieve an explicit agreement, whether the technology serves to help or hinder student performance. Therefore, it is essential to further investigate the long-term implications for learning and *how* the processes of cursive writing, typewriting, and drawing are working in the brain within a developmental perspective.

Cursive writing is a complex and central cultural skill (Kersey and James, 2013; Kiefer et al., 2015), involving many brain systems and the integration of both motor and perceptual skills (Vinci-Booher et al., 2016; Thibon et al., 2018). The skill of cursive writing is often used as a tool for learning (Arnold et al., 2017), considering the depths of processing that note-taking by hand provides, even in the absence of a review of the notes (Kiewra, 1985). Thus, cursive writing has been considered an essential precursor for further academic success (Fears and Lockman, 2018), and the skill is typically acquired during childhood in societies with a strong literacy tradition (Kiefer et al., 2015). Children must learn how to coordinate their hand movements accurately and produce the shape of each letter, and they may take several years to master this precise skill (Van der Meer and Van der Weel, 2017).

Today, most adults write using a keyboard and computer (Longcamp et al., 2005, 2006), and in some countries programs for elementary school education, typewriting on digital devices has already replaced traditional handwriting (Kiefer et al., 2015). Therefore, the amount of time spent writing by hand has been reduced as learning activities are increasingly relying upon digital devices (Mueller and Oppenheimer, 2014; Vinci-Booher et al., 2016). These devices (e.g., tablets and mobile phones) may improve a student’s ability to take notes, but they may also hinder learning in different and unknown ways (Stacy and Cain, 2015). Most educators acknowledge note-taking as an important factor of classroom learning (Stacy and Cain, 2015), and keyboard activity is now often recommended as a substitute for early handwriting as this type of activity is less demanding and frustrating for children (Cunningham and Stanovich, 1990).

Proponents of computers in the classroom stress the benefits of children being able to produce large texts earlier and receiving immediate feedback on their texts and questions through the Internet (Hultin and Westman, 2013). On the other hand, critics of computers in the classroom have found computer use to have a negative impact on course grades (Patterson and Patterson, 2017), lower class performance (Fried, 2008) as well as being distracting in the way that students habitually multitask (Sana et al., 2013). Compared to typewriting training, handwriting training has not only been found to improve spelling accuracy (Cunningham and Stanovich, 1990) and better memory and recall (Longcamp et al., 2006; Smoker et al., 2009; Mueller and Oppenheimer, 2014), but also improved letter recognition (Longcamp et al., 2005, 2008). These benefits have not only been found in traditional handwriting using an ink pen, but also in handwriting using a digital pen (Osugi et al., 2019). These results suggest that the involvement of the intricate hand movements and shaping of each letter may be beneficial in several ways. Therefore, the next question might be if *any* motor activity facilitates learning, or if the keyboard and pen cause different underlying neurological processes within the brain. If so, changing the motor condition while children are learning may affect their subsequent performance (Longcamp et al., 2005).

From the sensorimotor point of view, cursive writing and typewriting are two distinct ways of writing and may as well involve distinct processes in the brain (Longcamp et al., 2005, 2006; Alonso, 2015). The process of cursive writing involves fine coordination of hand movements when producing the shape of each letter, whereas typewriting requires much less kinesthetic information (Longcamp et al., 2006; Smoker et al., 2009; Kiefer et al., 2015). Several fMRI-studies, in preliterate (James and Engelhardt, 2012) and preschool children (e.g., James, 2010, 2017; Vinci-Booher et al., 2016), as well as adults (Menon and Desmond, 2001; Longcamp et al., 2003), have shown that areas related to writing processes are also activated when simply perceiving visual letters, suggesting that writing and reading are interrelated processes including a sensorimotor component (Longcamp et al., 2005, 2006).

Even though several researchers have pointed to certain task-specific brain areas, recent findings in modern neuroscience suggest that the brain is not that simple. Neural processes are highly dynamic (Lopes da Silva, 1991; Singer, 1993) and we still know very little about *how* the different brain systems are working together (Buzsáki, 2006). As recent findings of cognitive neuroscience have found processes in the brain to occur every millisecond, the EEG technique lends itself well to studying brain electrical activity as a function of cursive writing, typewriting, and drawing. The EEG-technique allows us to investigate changes in the state of the underlying networks (Lopes da Silva, 1991), and can reveal the continuously changing task-specific spatial patterns of activations (Pfurtscheller et al., 1996). Studies of cortical oscillations have become a fundamental aspect of modern systems neuroscience, yet, there are still conflicting definitions regarding the different rhythms and their cognitive usefulness (Fröhlich, 2016).

In general, brain oscillations are interactions between the thalamus and cortex and can be viewed as generated by changes in one or more parameters that control oscillations in neuronal networks (Pfurtscheller and Lopes da Silva, 1999). The complex interactions and the following distinctive frequencies are, in short, reflecting different cognitive processes (Klimesch et al., 1994; Berens and Horner, 2017). At the neural level, cortical oscillations have been found to reflect periodically membrane voltages that interact by synaptic transmission, reflecting a pattern of depolarization and hyperpolarization that enables or disables effective translation of incoming synaptic input into postsynaptic action potential firing (Fröhlich, 2016). In other words, the frequencies of the following oscillations depend both on the individual neurons and the strength of the action potentials (Lopes da Silva, 1991; Singer, 1993). This temporal organization of neural firing is of high importance and is also thought to be critical for the formation of long-term memories in the hippocampus (Berens and Horner, 2017).

Frequency-specific changes in the ongoing EEG, that are not phase-locked to a specific event, can be observed in form of event-related synchronization (ERS) (an increase in spectral amplitude) or event-related desynchronization (ERD) (a decrease in spectral amplitude) (Pfurtscheller and Aranibar, 1977; Pfurtscheller and Lopes da Silva, 1999). These longer-lasting ongoing changes can be detected using spectral analyses (Klimesch, 1996), e.g., induced temporal spectral evolution (TSE), to study differences in a given frequency band (Pfurtscheller et al., 1994; Salmelin and Hari, 1994). The TSE technique calculates temporal dynamics of EEG oscillations and quantifies both event-related suppressions and/or enhancements of rhythms after the original EEG-data have been inspected and filtered through specific filters (Salmelin and Hari, 1994). Both ERD and ERS are highly frequency-specific and can be displayed in both the same or different locations on the scalp simultaneously (Lopes da Silva, 1991; Pfurtscheller, 1992; Pfurtscheller et al., 1996; Pfurtscheller and Lopes da Silva, 1999).

In a recent EEG-study, Van der Meer and Van der Weel (2017) found that drawing by hand activates larger networks in the brain compared to typewriting, and concluded that the involvement of fine hand movements in note-taking, as opposed to simply pressing a key on a keyboard, may be more beneficial for learning, especially when encoding new information. They found a desynchronized activity within the alpha band in the parietal and occipital areas of the brain, suggesting this activity to be beneficial for learning, especially as the activity was shown to occur in the rather deep structures of the brain (e.g., hippocampus, the limbic system). Both handwriting and drawing are complex tasks that require integration of various skills (Van der Meer and Van der Weel, 2017), and adults often use the same term to refer to young children’s writings and drawings (Treiman and Yin, 2011). Both processes involve several visuomotor components and precise coordination (Planton et al., 2017) to produce artificial marks that appear on a surface (Treiman and Yin, 2011). As drawing can be said to be just as complex as handwriting, this activity is not used daily as an intensive learning strategy in the form of written productions (Planton et al., 2017). Nevertheless, drawing may exhibit just as much higher-level processing as handwriting, if not more so, especially when it comes to creating creative drawings as opposed to writing standardized letters. Therefore, it would be interesting to investigate whether drawing and cursive writing engage similar or different activation patterns in the brain, and how they differ from typewriting on a keyboard based on the literature mentioned above.

As previous studies have found support for the benefits of note-taking by hand in terms of learning, the present study aimed to expand the findings by Van der Meer and Van der Weel (2017), and further investigate the neurobiological differences in the adult and child brain related to cursive writing, typewriting, and drawing, using high-density EEG. It was hypothesized that handwriting and drawing would activate similar brain areas, in profound structures of the parietal lobe, to a greater extent than typewriting on a keyboard. Studying the adult brain state can provide valuable information (Vinci-Booher et al., 2016), but investigating the stages that lead to the adult-like neural signatures can help us better understand cognitive development and why the brain responds to certain stimuli the way it does as a result of experience (James, 2010). Therefore, the present study includes a group of 12-year-old children, in addition to adults, to investigate if the same activations are apparent as in the literate adult, and perhaps even more critical in terms of learning and initiation of essential neuronal structures in the brain. Hence, the present study aims to investigate the importance of teaching cursive writing in school and to further explore which strategies of cursive writing, typewriting, or drawing are more beneficial to facilitate and optimize learning in the classroom.

## Materials and Methods

### Participants

Sixteen healthy school-aged children and sixteen healthy adults were recruited to participate in this study at the Developmental Neuroscience Laboratory at NTNU (Norwegian University of Science and Technology). The study followed a cross-sectional design to study differences in oscillatory brain activity in tasks of cursive writing, typewriting, and drawing among children and adults. The school-aged children were recruited from 7th graders at the Waldorf school in Trondheim, who are very used to cursive handwriting and drawing. Interested parents contacted the lab for further information about their child’s participation. The adults were recruited through different lectures at the university campus, or they were contacted through friends. All participants were right-handed, as determined by the Edinburgh Handedness Inventory (Oldfield, 1971). Only right-handed participants with a handedness quotient larger or equal to +0.6 took part in the study, ranging from lowest to highest, 0.65–0.93 in adults and 0.60–1.00 in children, respectively. Four of the children were removed from further analysis due to inadequate data or other information that could affect the data analyses (e.g., dyslexia, ADHD, or prematurity). In addition, four of the adults were removed due to inadequate data and to maintain equal sized groups. Because of this, the resulting total sample included 12 school-aged children and 12 young adults.

For the school-aged children (four boys and eight girls), the mean age was 11.83 years (*SD* = 0.39). Parents gave their informed consent concerning their children, and the child could withdraw from the experiment at any time without any consequences. For the adults (six men and six women), the mean age was 23.58 years (*SD* = 2.02). The adults also gave their informed consent and could withdraw at any time. The adults were rewarded with a 150 NOK cinema ticket, whereas the school-aged children were rewarded with snacks in the lab and a picture of themselves with the EEG-net on. The Regional Committee for Medical and Health Ethics approved the study.

### Experimental Stimuli and Paradigm

Psychological software tool, E-prime 2.0, was used to generate 15 different Pictionary words on a separate Microsoft Surface Studio. The participants used a digital pen to write in cursive by hand and draw directly on the touch screen, and a keyboard to typewrite the presented words. The screen measured 25.1″ × 17.3″ × 0.5″ and had a screen resolution of 4500 × 3000 (192 PPI) pixels.

**FIGURE 1:**
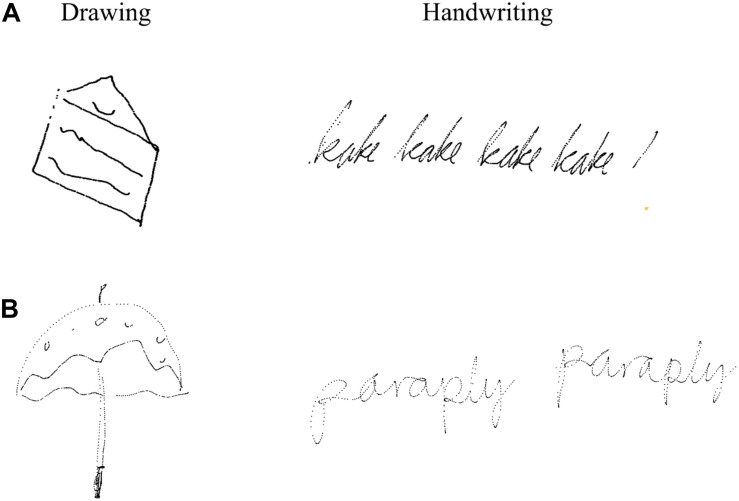
Example of writings and drawings of **(A)** 12-year-old boy and **(B)** 23-year-old female student. Figure is reproduced using x,y-coordinates over time from the touchscreen.

The experiment included a total of 45 trials, where each word was presented in three different conditions, represented in a semi-randomized order. The 15 words varied in difficulty, from concrete words, such as “shoe,” to more abstract words, such as “birthday.” For each trial, participants were instructed to either (a) *write in cursive* the presented word with a digital pen directly on the screen, (b) *type* the presented word using the right index finger on the keyboard, or (c) *draw* the presented word by freehand with a digital pen directly on the screen. Whereas handwriting and typewriting were both relatively simple transcription tasks, drawing included higher-level processing (ideation). Before each trial, an instruction appeared 1–2 s before one of the 15 target words appeared, and the participants were given 25 s to either handwrite, type, or draw the word. EEG data were recorded only during the first 5 s of each trial. The participants could draw and write wherever they preferred directly on the screen. The words that were typed were the only words that did not appear on the screen while the participant was typewriting. A small sound indicated that the current trial was over and a new one was about to start. The drawings and writings produced by the participants were stored for offline analyses (see Figure 1).

### EEG Data Acquisition

An EEG Geodesic Sensor Net (GSN) (Tucker, 1993; Tucker et al., 1994) with 256 evenly distributed sensors was used to record EEG activity from the participant’s scalp. The signals were amplified using a high-input EGI amplifier, at maximum impedance at 50 kΩ to ensure optimal signal-to-noise ratio (Picton et al., 2000; Ferree et al., 2001). The amplified signals were recorded by Net Station software with a sample rate of 500 Hz. All data were stored for further off-line analyses.

### Procedure

Participants usually arrived several minutes prior to the experiment. On arrival, a consent form with all necessary information was given to the participants to sign. For the children, both the parent and the child signed the consent form. The participant’s head was measured to find the correct size for the net. While the participant completed the Edinburgh Handedness Inventory (Oldfield, 1971), the net was soaked in a saline electrolyte for 15 min to optimize electrical conductivity. After being partially dried from the soaking, the net was mounted on the participant’s head. Next, the participant was moved to the experimental room where further information regarding the experiment was given. The experimental room was separated from the control room, where two assistants operated the computers necessary for data acquisition. The participant was sitting comfortably in an adjustable chair in front of a table with two levels, to minimize unnecessary movement in between trials that could cause artifacts in the data. A pillow was used to avoid tension in the back, and the table with the screen (on the second level) was placed as close as possible to the participant. A keyboard was further placed (on the nearest level) in a preferred position for the participant, and a digital pen was used for writing and drawing on the screen. The participants were asked to support their elbow to minimize hand movements in the trials using the pen. In addition, they were asked to sit as still as possible, while at the same time trying to perform the tasks as naturally as possible. The EEG-net was connected to the amplifier and the impedance of the electrodes was checked. Electrode connectivity could be improved by either adjusting their position or by adding additional saline electrolyte for better contact.

A pre-test was completed before the experiment where one of the assistants was present in the room. During this test, the participants could ask questions if needed, and necessary adjustments could be made. The pre-test included one example of each experimental condition, using a word not included in the actual experiment. The experiment started immediately after the pre-test was finished, the impedance was approved, and the participant was ready.

Two experiments were conducted at the same time, with a total of six different conditions, resulting in a total of 90 trials. In order to tap into the neural underpinnings of creative processes, the additional conditions in the separate experiment included (d) *describe* the presented word with a digital pen directly on the screen (e), *copy* the presented sentence with a digital pen directly on the screen, and (f) *draw a copy* of the presented drawing with a digital pen directly on the screen. However, the focus of the present paper was on comparing neuronal oscillations during the paradigm tasks of handwriting, typewriting, and drawing. Data acquisition was carried out in two blocks (45 trials in each) and lasted for about 45 min. Between the two blocks, the participants were given a pause where they could drink water and have a break from the screen. A pause was also initiated if the participant was moving a lot or appeared nervous, to remind the participant to relax and sit as still as possible. Further, the participants were told to knock on the window, separating the experimental room and control room, if they needed additional breaks or had any questions during the experiment.

### Data Pre-analyses

Brain Electrical Source Analysis (BESA) research software version 7.0 was used to analyze the EEG data. Recordings were segmented using Net Station software and then exported as raw files with the appropriate auxiliary files attached, prior to the analyses in BESA. Average epoch was set to −250 to 4500 ms with a baseline definition of −250 to 0 ms. Low cut-off filter was set to 1.6 Hz to remove slow drift in the data, while the high cut-off filter was set to 75 Hz. The notch filter was set to 50 Hz to avoid line interference in the data.

Artifact contaminated channels, caused by head or body movements, were either removed or interpolated using spherical spline interpolation (Perrin et al., 1989; Picton et al., 2000). A maximum limit of 10% of the channels could be defined as bad. When scanning for artifacts, threshold values for gradient, low signal, and maximum amplitude were set to 75, 0.1, and 200 μV, respectively. Manual artifact correction was applied to separate important brain activity from artifacts using manual and semi-automatic artifact correction with fitting spatial filters (Berg and Scherg, 1994; Ille et al., 2002; Fujioka et al., 2011). When it was not possible to apply manual artifact correction, an automatic artifact correction (with values 150 μV for horizontal and 250 μV for vertical electrooculogram amplitude thresholds) was applied to explain artifact topographies by principal component analysis (PCA) (Ille et al., 2002).

For the school-aged children, the mean numbers of accepted trials were 11 (*SD* = 1.63) for handwriting, 9.67 (*SD* = 2.74) for typewriting, and 12.08 (*SD* = 1.89) for drawing, respectively. For the adults, the mean numbers of accepted trials were 14.33 (*SD* = 0.98) for handwriting, 13.42 (*SD* = 1.24) for typewriting, and 14.08 (*SD* = 1.56) for drawing, respectively. After all the data were sufficiently artifact-free, time-frequency analysis in brain space was performed.

### Time-Frequency Analysis in Brain Space

**FIGURE 2:**
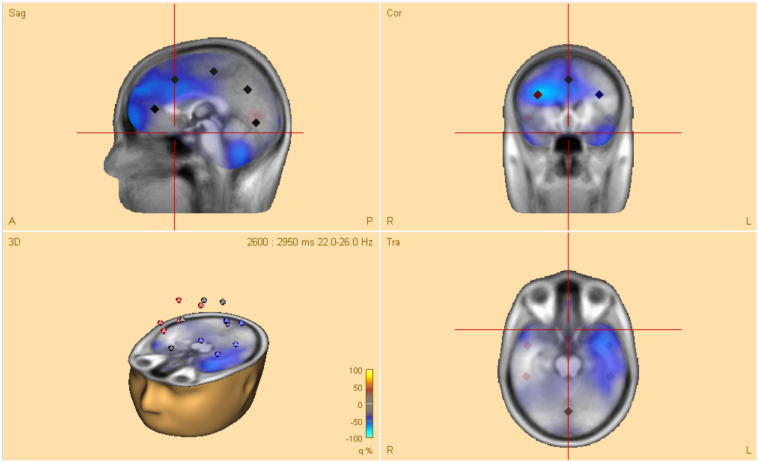
Head model of a typical 12-year-old boy. The model shows four dipoles (with location and direction of electrical current) in regional sources of interest, over frontal, central, temporal parietal, as well as occipital areas.

Time-frequency analysis in brain space was conducted for analysis of oscillatory activity, using multiple source dipoles that modeled the main brain regions of interest (see Figure 2). As the EEG-technique measures voltage changes at the scalp around dipoles, the orientations of these dipoles are essential as they provide the specific distribution of an EEG-activity (Luck, 2005; Fröhlich, 2016). Measuring oscillatory activity directly on scalp surface electrodes may not be ideal, due to mixed brain source contributions and wide distribution of focal brain activity on the scalp surface caused by the nature of dipole fields and the smearing effect of volume conduction in EEG. Therefore, optimal separation of brain activity was achieved using source montages derived from a multiple source model where waveforms separated different brain activities (Scherg and Berg, 1991). The multiple source model transforms the recorded data from sensor level into brain source space and provides source waveforms that can be used as a direct measure for the activity in the brain regions of interest on a single trial basis (Hoechstetter et al., 2004). A discrete multiple source modeling was used for the time-frequency transformation. This model is created from averaged ERP data and/or sources in the brain regions of interest and is used to create an inverse spatial filter, i.e., a source montage that separates the different brain activities. The source model is then used to calculate a source montage and the source waveforms of the single trials. The regional sources of interest included the frontal, central, temporal, parietal, and occipital areas (see Figure 2). Using the procedure of multiple source model, it is possible to separate the time-frequency content of different brain regions even if their activities severely overlap at the surface of the scalp (Hoechstetter et al., 2004).

A 4-shell ellipsoidal head model (Berg and Scherg, 1994; Hoechstetter et al., 2004), was used to analyze the sources of interest of the young adults after loading the artifact-corrected coordinate files. The values for bone thickness and conductivity were set to 7.0 and 0.0042 mm (default values in BESA), respectively. For the 12-year-old children, age-appropriate template models were set to 12 years for realistic templates for source analysis.

The time-domain signal was transformed into the time-frequency domain by selecting a certain temporal resolution using complex demodulation (Papp and Ktonas, 1976). The time-frequency displays, representing changes in amplitude over time (TSE, temporal spectral evolution), were generated from each single trial by averaging spectral density amplitudes over trials such that each graph displayed, plotted the spectral amplitude density of one montage channel over time and frequency which were normalized to the baseline for each frequency (Pfurtscheller et al., 1994, 1996; Hoechstetter et al., 2004). Average evoked response signals were subtracted to focus only on induced (instead of evoked) brain activity before computing the TSE (Pfurtscheller et al., 1994; Handy, 2005).

A time-frequency display is shown where the power/amplitude for each time is normalized to the mean power/amplitude of the baseline epoch for that frequency. The x-axis shows the time relative to the event, the y-axis shows the frequencies. The intensities are displayed as a color-coded plot. The resulting value is computed as:

$$\text{TSE}=\frac{\text{A}\left(t,f\right)-{\text{A}}_{\text{baseline}}\left(f\right)}{{\text{A}}_{\text{baseline}}\left(f\right)}. 100\%$$

with A(*t*,*f*) = activity at time *t* and frequency *f* (either power or absolute amplitude) and A_baseline_(*f*) = mean activity at frequency *f* over the baseline epoch. The TSE value is in the range from [−100%, + ∞] and describes the spectral change of activity at sampling time *t* relative to the activity during the baseline epoch. A value of +100% means that activity is twice as high as during the baseline epoch.

Comparisons between the three conditions handwriting, typewriting, and drawing were computed for each participant with time-frequency displays (changes in amplitude over time). TSE displays were limited between frequency cut-offs of 4–60 Hz, while frequency and time sampling were set at 1 Hz and 50 ms.

### Statistical Analyses

Probability of significance in amplitude values and frequency ranges between each of the three conditions was tested with BESA Statistics 2.0. Using this program, average TSE statistics for each participant could be computed to use these significant time-frequency ranges as guides in finding maximum oscillatory activity in the individual TSEs. To address the multiple comparisons problem, a combination of permutation tests and data clustering was employed in the statistical test. Data clusters that showed a significant effect between conditions were assigned initial cluster values. Using both between-groups and within-group ANOVA’s, these initial cluster values were passed through permutation and assigned new clusters so that the significance of the initial clusters could be determined. A Bonferroni correction was used to adjust for multiple comparisons (Simes, 1986). Cluster alpha (the significance level for building clusters in time and/or frequency) was set at 0.01, and the number of permutations was set at 10.000. Low- and high cut-offs for frequency were kept at 4 and 60 Hz, and epochs were set from −250 to 4500 ms. *post-hoc* tests were run to test for statistical differences between the three conditions and two age groups.

## Results

### Individual Time-Frequency Responses

**FIGURE 3:**
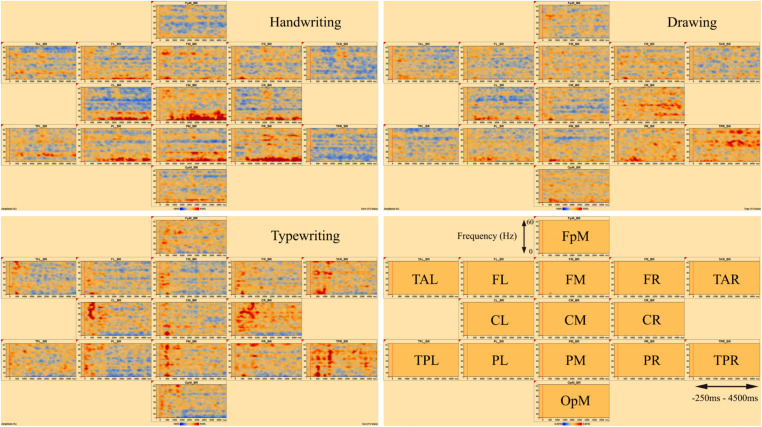
Individual time-frequency displays of a typical male adult. The y-axes display frequencies from 4 to 60 Hz. The x-axes display the time interval from baseline to 4500 ms of recordings of the trial. The signal magnitude (amplitude%) reflects the estimated neural activity in the various brain regions during the experimental conditions compared to baseline activity (−250 to 0 ms). Event-related synchronization (ERS) is shown as red-colored contours, more prominent in lower frequencies (theta 4–8 Hz) for handwriting and drawing and higher frequencies (beta 12–30 Hz and gamma >30) for typing. Event-related desynchronization (ERD) is shown as blue-colored contours, more prominent in higher frequencies (beta 12–30 Hz and gamma >30) for handwriting and drawing and lower frequencies (theta 4–8 Hz) for typing. Brain areas included the following frontal, temporal, central, parietal and occipital areas: FpM, fronto-polar midline; FL, frontal left; FM, frontal midline; FR, frontal right; TAL, temporal anterior left; TAR, temporal anterior right; TPL, temporal posterior left; TPR, temporal posterior right; CL, central left; CM, central midline; CR, central right; PL, parietal left; PM, parietal midline; PR, parietal right; OpM, occipito-polar midline.

**FIGURE 4:**
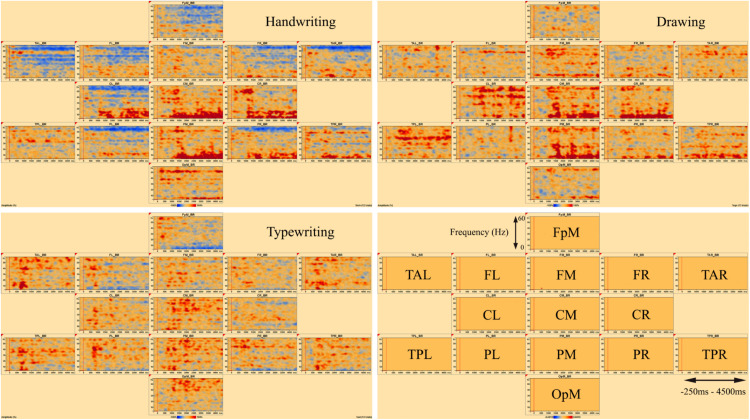
Individual time-frequency displays of a typical 12-year-old girl, in frontal, temporal, central, parietal and occipital areas. The y-axes display frequencies from 4 to 60 Hz. The x-axes display the time interval from baseline to 4500 ms of recordings of the trial. The signal magnitude (amplitude%) reflects the estimated neural activity in the various brain regions during the experimental conditions compared to baseline activity (−250 to 0 ms). Event-related synchronization (ERS) is shown as red-colored contours and event-related desynchronization (ERD) is shown as blue-colored contours, showing the same activation patterns as for the adult in Figure 3.

Figures 3, 4 display the results of individual TSE (temporal spectral evolution) maps of brain regions of interest for the three experimental conditions handwriting, typewriting, and drawing, for a typical adult and child participant. Brain regions of interest included frontal, temporal, parietal, central as well as occipital areas, in frequencies from theta (4 Hz) and up to gamma (60 Hz) range. The signal magnitude (amplitude%) reflects estimated neural activity in the various brain regions compared to baseline (−250 to 0 ms) activity. Increased spectral amplitude [induced synchronized activity, event-related synchronization, (ERS)] is shown as red-colored contours and decreased spectral amplitude [induced desynchronized activity, event-related desynchronization (ERD)], is shown as blue-colored contours.

In the parietal and central areas, event-related synchronization (ERS) was more prominent in lower frequencies (theta 4–8 Hz) for handwriting and drawing, as opposed to in higher frequencies (beta 12–30 Hz, and gamma > 30 Hz) for typewriting. For handwriting, this activity appeared around 500–1000 ms and lasted throughout the trial in both adults and adolescents. For drawing, however, this activity appeared around 500 ms and lasted, though to a lesser extent, throughout the trial in the adults, as opposed to the children, where it appeared around 1000 ms and lasted consistently throughout the trial. For typewriting, this activity appeared to vary from 0 to 500 ms in both beta (12–30 Hz) and gamma (>30 Hz) frequencies in both adults and children. As for event-related desynchronization (ERD), this activity was more prominent in higher frequencies (beta 12–30 Hz, and gamma > 30 Hz) for handwriting and drawing and in lower frequencies (theta 4–8 Hz and, to a lesser extent, alpha 8–12 Hz) for typewriting. For handwriting and drawing in both groups, ERD activity appeared around 0 ms and lasted throughout the trial. In contrast, for typewriting, it appeared around 1000 ms and lasted throughout the trial for adults, whereas for children the activity was more variable and took place from 500 to 1500 ms. Figures 3, 4 show the individual TSE maps of the brain regions of interest in a typical adult and child, respectively. These patterns were largely consistent among the participants in both groups.

### Main Effects and *post-hoc* Analyses

Statistical analyses were run to test for statistical differences between the conditions and groups. Tables 1, 2 display the detailed main effects (within-group ANOVA) of the permutation results (of clusters where the null hypothesis is rejected, i.e., data are not interchangeable) of the adults and children, respectively. These results revealed 10 and 4 significant clusters for the adults and the children, respectively.

**TABLE 1:** Permutation test of adult results for 10 significant clusters in decreasing order.

Cluster ID	*p*-value	Cluster value	Mean for type	Mean for draw	Mean for handwrite	Start time	End time	Start frequency	End frequency
TPL	0.0009	1378	−0.32	−0.07	−0.08	2100	4500	4	8
PR	0.0026	1168	−0.23	−0.11	0.21	2350	4050	4	11
PM	0.0034	1055	−0.12	0.06	0.34	2400	4000	4	9
PR	0.0068	833	−0.26	−0.05	0.14	1200	2500	4	8
CL	0.0084	769	−0.18	−0.13	0.22	3700	4500	4	15
PL	0.0116	707	−0.22	−0.09	0.25	2750	3550	4	12
TPR	0.0141	664	−0.30	−0.03	−0.03	2800	3650	4	11
PL	0.0141	660	−0.22	−0.07	0.25	3650	4500	4	10
PL	0.0264	560	−0.20	−0.06	0.19	1200	1950	4	12
CM	0.0345	509	−0.03	−0.04	0.34	3800	4400	6	14

*TPL, temporal parietal left; PR, parietal right; PM, parietal midline; CL, central left; PL, parietal left; TPR, temporal parietal right; CM, central midline.*

**TABLE 2:** Permutation test of child results for four significant clusters in decreasing order.

Cluster ID	*p*-value	Cluster value	Mean for type	Mean for draw	Mean for handwrite	Start time	End time	Start frequency	End frequency
TPR	0.0000	3746	−0.33	0.27	0.01	1150	4500	4	13
PL	0.0125	794	−0.24	0.20	−0.03	1950	3050	5	16
PR	0.0154	726	−0.36	−0.02	0.02	3000	3850	4	16
PL	0.0411	518	−0.36	−0.02	−0.09	3800	4500	4	8

*TPR, temporal parietal right; PL, parietal left; PR, parietal right.*

**FIGURE 5:**
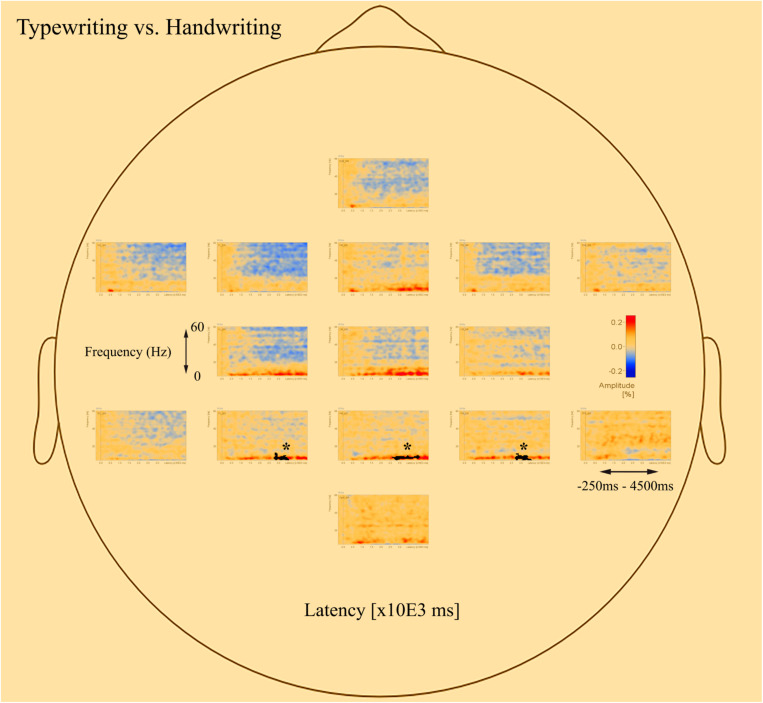
Head model (nose up) with average significant (*p < 0.05) data clusters in the various sources of interest when handwriting is compared to typewriting in all adults. Three significant clusters (marked in black) were found in the parietal left (PL), parietal midline (PM), and parietal right (PR). For handwriting, an event-related synchronized activity in the theta (4–8 Hz) range is apparent in parietal, central, occipital, as well as in frontal areas. Event-related desynchronization is apparent in the beta (12–30 Hz) and gamma (>30 Hz) range in the central and frontal areas.

**FIGURE 6:**
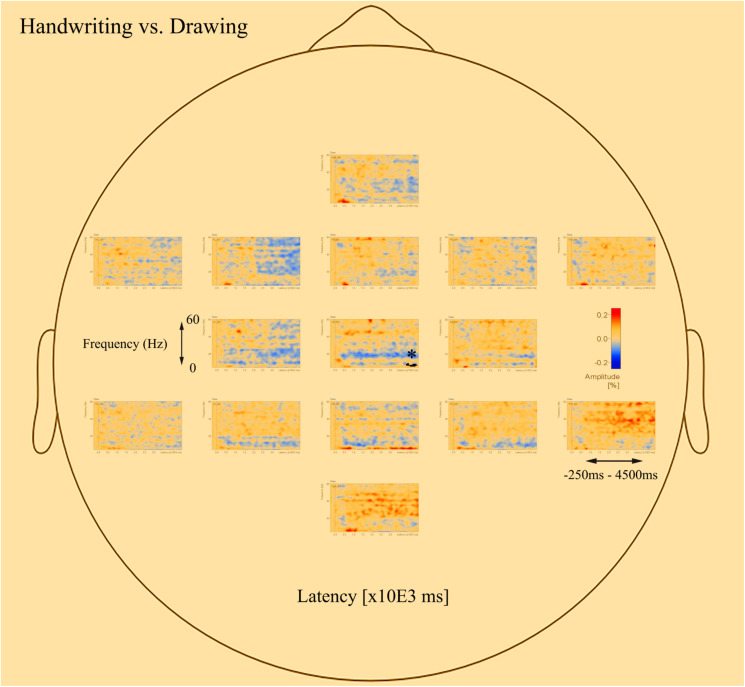
Head model (nose up) with average significant (*p < 0.05) data clusters in the various sources of interest when drawing is compared to handwriting in all adults. One significant cluster (marked in black) was found in the central midline (CM). For drawing, areas in the parietal and central regions are dominated by a desynchronized activity in the alpha (8–12 Hz) and beta (12–30 Hz) range. In addition, event-related synchronization is apparent in the theta (4–8 Hz) range in the parietal midline (PM).

The *post-hoc* tests revealed significant differences in oscillatory activity primarily in the alpha (8–12 Hz) and theta (4–8 Hz) band between handwriting, typewriting, and drawing among both age groups. As the differences between typewriting and drawing, in both children and adults, were similar to the differences between typewriting and handwriting, only the statistical differences between typewriting and handwriting, and handwriting and drawing in the adults are reported here. Further investigations of the parietal and central brain areas in both age groups were conducted to study the various brain activation patterns of the different learning strategies. Figures 5, 6 display the *post-hoc* results of the permutation tests in the adults between handwriting and typewriting, and between handwriting and drawing, respectively. When handwriting was compared to typewriting, the permutation results showed three significant positive clusters (in black), in the parietal right (PR), parietal midline (PM), and parietal left (PL) areas (see Figure 5). When handwriting was compared to drawing, the results showed one significant positive cluster (in black), in the central medial (CM) area (see Figure 6). These positive clusters suggest separate processes (differences in band power) between handwriting and typewriting in the parietal areas, as well as separate processes between handwriting and drawing in the central midline area.

The significant clusters of differences in band power were found mainly in the parietal and central regions. The parietal areas of the brain have been associated with cognitive processing of language and mechanisms for attention (e.g., Pfurtscheller et al., 1994; Brownsett and Wise, 2010; Benedek et al., 2014), whereas the central areas are influenced by the somatosensory cortex (e.g., Velasques et al., 2007). Therefore, these areas were chosen to further focus on the underlying brain electrical activity as a function of handwriting, typewriting, and drawing. Additionally, the potential deep structures of the brain, that may have their beneficial effects on learning (Van der Meer and Van der Weel, 2017), may be found in these areas.

**FIGURE 7:**
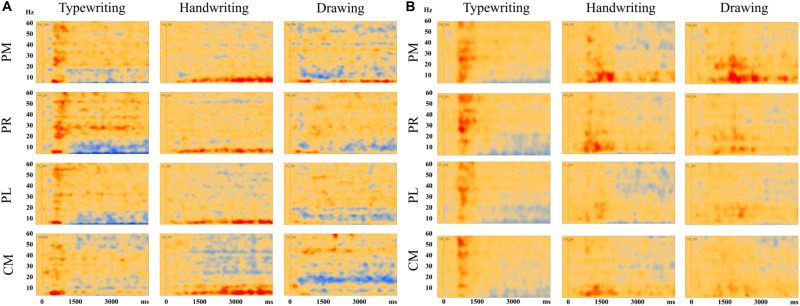
Average results of all participants for typewriting, handwriting, and drawing in **(A)** adults and **(B)** children, in the parietal and central regions: PM, parietal midline; PR, parietal right; PL, parietal left; CM, central midline. For the adults, these areas showed event-related synchronization (ERS) in the theta (4–8 Hz) range for handwriting and event-related desynchronization (ERD) activity in the theta (4–8 Hz) and, to a lesser extent, in the alpha (8–12 Hz) range for typewriting. For drawing, event-related synchronization (ERS) was apparent in the theta (4–8) range in parietal midline as for handwriting. In addition, event-related desynchronization (ERD) activity was apparent in the alpha (8–12 Hz) and beta (12–30 Hz) range. The same patterns were observed, though to a lesser extent, in the children.

Figure 7 displays the average of all participants for handwriting, typewriting, and drawing in adults (see Figure 7A) and children (see Figure 7B) in the central and parietal brain regions of interest. For adults, handwriting appeared to be dominated by an event-related synchronization (ERS) (red areas) in the theta (4–8 Hz) range, in addition to an event-related desynchronization (ERD) activity in the beta (12–30 Hz) and gamma (>30) range. The theta activity appeared around 1000 ms and lasted throughout the trial. Contrary to handwriting, typewriting appeared to be dominated by an event-related desynchronized (ERD) (blue areas) activity in the theta (4–8 Hz) range and, to a lesser extent, in the alpha (8–12 Hz) range. This activity appeared around 1500 ms and lasted throughout the trial. In drawing, a synchronized theta (4–8 Hz) activity was apparent in the parietal midline (PM) and the parietal right (PR), in addition to a desynchronized alpha (8–12 Hz) and beta (12–30 Hz) range activity from around 500 ms and throughout the trial (see Figure 7A).

The same tendencies could be observed for the children, but they were less evident compared to the adults (see Figure 7B). For the children, desynchronized and synchronized theta (4–8 Hz) range activity was also apparent in typewriting and to a lesser extent in handwriting, respectively. In drawing, synchronized theta (4–8 Hz) range activity was also apparent, yet to a smaller degree, in parietal midline (PM) and parietal right (PR). In addition, a desynchronized activity appeared to dominate in the gamma (>30 Hz) range in handwriting for the children.

## Discussion

The aim of the current study was to further investigate brain electrical activity as a function of handwriting, typewriting, and drawing using high-density EEG in 12-year-old adolescents and adults. Fifteen different words, varying in task difficulty, were visually presented on a screen and the participants used a digital pen to write and draw directly on the touch screen, and a keyboard to type the presented words. Whereas handwriting and typewriting were both relatively simple transcription tasks, drawing included higher-level processing. TSE analyses were performed to explore underlying differences in brain oscillatory activity when participants were using a keyboard vs. a pen. In addition, the present study aimed to explore if drawing and cursive writing are activating similar or different processes within the brain. Regional sources of interest included frontal, temporal, parietal, central as well as occipital areas, in frequencies from theta (4 Hz) and up to gamma (60 Hz) range. Induced desynchronization is often taken to be an electrophysiological correlate of activated cortical areas involved in the processing of perceptual or cognitive information, or in the production of motor behavior (Pfurtscheller, 1992). To focus on oscillatory brain activity in specific frequency bands that has shown to have beneficial effects on learning and memory (Pfurtscheller and Lopes da Silva, 1999), the parietal and central areas were further investigated. These areas have also been associated with cognitive processes in visual perception (e.g., Pfurtscheller et al., 1994; Vilhelmsen et al., 2019) and language (e.g., Brownsett and Wise, 2010; Benedek et al., 2014) as well as to be influenced by sensorimotor cortex (e.g., Velasques et al., 2007).

### TSE – Individual Analyses

The present findings revealed differences in oscillatory activity between handwriting, typewriting, and drawing for both children and adults. By visually reviewing the individual TSE analyses of a typical participant in both groups, these differences are shown as changes in band power (increase or decrease in spectral amplitude) between handwriting, typewriting, and drawing, apparently representing different sensorimotor processes within the brain. However, there seem to be more similarities between handwriting and drawing, compared to typewriting, despite differences in task difficulties, thus supporting the study by Van der Meer and Van der Weel (2017).

### Synchronized Theta Activity in Parietal and Central Areas in Handwriting

Event-related synchronization within the theta (4–8 Hz) band has been found to correlate with working memory performance and the ability to encode new information (Klimesch et al., 1994, 1996, 2001; Klimesch, 1999; Raghavachari et al., 2001; Clouter et al., 2017). Therefore, our findings seem to support the potential benefits of handwriting activity for learning. Although the handwriting task in the present study was a relatively simple transcription task, it was still evident that the observed oscillatory brain activity is present whenever the specific sensory-motor movements involved in handwriting practices are included. Even though participants did not take personal notes from a lecture as in a natural classroom environment, it still seems this type of oscillatory activity in the brain is present when writing letters by hand or when drawing, as opposed to when simply pressing a key on the keyboard. Klimesch et al. (1994) have also proposed that hippocampal activity is reflected within the theta band and shown as synchronized theta band power. However, this activity can be difficult to pick up with EEG, yet it is likely that the present activity stems from the rather deep structures of the brain (e.g., hippocampus and the limbic system) and adds further support for handwriting and its relation to optimized learning.

Moreover, Bland and Oddie (2001) have found support for synchronized theta activity in mechanisms underlying sensorimotor integration. Although the present study does not replicate the desynchronized activity in the alpha band found by Van der Meer and Van der Weel (2017), it still supports their findings because both ERS and ERD are highly frequency-specific, i.e., the alpha and theta band respond in different and opposite ways (Pfurtscheller et al., 1996; Pfurtscheller and Lopes da Silva, 1999). In terms of cognitive effort, where the alpha band desynchronizes, the theta band synchronizes. Therefore, theta synchronization may indicate that different neural generators are involved, as with alpha desynchronization (Klimesch et al., 1994; Klimesch, 1999). Thus, our findings corroborate the findings by Van der Meer and Van der Weel (2017), but in a different frequency band. However, whereas alpha desynchronization is highly task-specific and correlates with (semantic) long-term memory performance, theta synchronization correlates with working memory performance and the ability to encode new information (Klimesch et al., 1994, 1996, 2001; Klimesch, 1999; Clouter et al., 2017).

Lower frequencies are ideal for enabling communication over longer distances in the brain. Several studies have found support for lower frequencies to “gate” the occurrence of faster oscillations, e.g., theta (4–8 Hz) oscillation in humans often gates the gamma (>30 Hz) oscillation (Canolty et al., 2006). For handwriting, especially in the individual TSE-analyses, a desynchronized gamma (>30 Hz) activity was apparent together with the synchronized theta (4–8 Hz) activity (see Figures 3, 4). In general, gamma oscillations appear to be underlying mechanisms of neural coding (Singer, 1993), and this theta-to-gamma cross-frequency coupling seems to be related to studies finding gamma networks to desynchronize and theta networks to synchronize during encoding, retrieval (Solomon et al., 2017), as well as during episodic memory formation (Burke et al., 2013). Solomon et al. (2017) have also suggested low-frequency oscillations to be essential for interregional communication in the human brain. However, other studies (e.g., Osipova et al., 2006), have found synchronized activity in both theta and gamma bands, thereby indicating that further research of this coupling is needed. Also, because of the broad definition of the gamma frequency (30–100 Hz), the present study only observed a small portion of the gamma band.

### Desynchronized Theta Activity in Parietal and Central Areas in Typewriting

Conversely, for typewriting, a desynchronized activity was evident in the theta (4–8 Hz) and, to a lesser extent, in the alpha (8–12 Hz) range. The lower alpha (8–10 Hz) range has been found to reflect non-task related cognitive processes, such as expectancy, lower attention, and alertness (Klimesch et al., 1992, 1994; Klimesch, 1999). Therefore, this finding could reflect the focus in finding the correct keys on the keyboard, typewriting with the index finger only, and not seeing the output appearing on the screen. The fact that the words produced by the participants did not appear on the screen may have affected the participants’ attention in trying to write as correctly as possible. Typewriting with only the index finger may also have been unfamiliar and could have contributed to the need for increased attention.

The finding of desynchronized activity in the upper alpha (10–12 Hz) range, on the other hand, has been found to correlate with increasing task demands (Boiten et al., 1992). Within the alpha band, a desynchronization seems to imply that the oscillators within the band are no longer coupled and start to oscillate with different frequencies (Klimesch, 1999), implying that more areas of the brain are activated and multiple processes are occurring (Basar et al., 2001). However, the desynchronized activity within the upper alpha (10–12 Hz) band observed here is apparent to a lesser extent, and is most likely due to increased attention and task demand because of the unfamiliar movements when typewriting with the index finger only. An alternative interpretation of this rhythm could also be the movement mu (8–12 Hz) rhythm. This rhythm appears to desynchronize during movement (Cruikshank et al., 2012). Whereas the participants were resting their elbow in the drawing and handwriting condition, thereby effectively reducing movement, more arm movements were present when they used the keyboard. However, since the theta, alpha and mu rhythms are nearby in frequencies, they may be difficult to distinguish from each other. Therefore, its relation to learning remains unclear.

### Different and Similar Activation Patterns in Handwriting and Drawing

The results reported above suggest that handwriting and drawing, just like typewriting and handwriting, are two separate processes within the brain. However, the neural processes involved in handwriting and drawing seem to be more similar to each other compared to typewriting. Our findings therefore both corroborate and extend the findings of Van der Meer and Van der Weel (2017). Compared to handwriting, drawing exhibited a desynchronized alpha (8–10 Hz) and beta (12–30 Hz) range activity. These findings suggest an increase in cognitive effort and attentive information processing (Lopes da Silva, 1991; Boiten et al., 1992), as well as the inclusion of motor actions (Pfurtscheller et al., 1996), most likely related to higher-level processing during the ideation phase when participants are figuring out exactly what to draw. In addition, the synchronized theta (4–8 Hz) band activity found in handwriting was also apparent in certain areas of the parietal regions. Therefore, as with handwriting, drawing seems to facilitate learning to encode new information. The synchronized theta band activity in the parietal regions seems to be activated both when producing letters by hand and when creating creative drawings.

Using a meta-analysis of brain imaging studies, Yuan and Brown (2015) suggested that handwriting and drawing might employ the same underlying sensorimotor networks, but that some differences exist between them in the parietal areas. The reason for this difference may not be surprising, considering the extensive involvement of language and letters in writing (Treiman and Yin, 2011), which drawing appears to lack. Although the present study only found a significant cluster in the central areas differentiating between handwriting and drawing, the average results clearly showed underlying differences in oscillatory activity in the parietal areas as well, especially in the alpha (8–10 Hz) and beta (12–30 Hz) range. The observed brain processes involved in handwriting and drawing seem to support the notion that both employ the same underlying sensorimotor networks.

As for the children, the same tendencies between handwriting, typewriting, and drawing could be observed, but they were far less evident compared to the adults. The reason for these less evident activation patterns could be due to more artifact-contaminated data in the children, resulting in fewer trials. EEG is particularly sensitive to movement, and young children are prone to movements. An alternative interpretation of these results may be that the oscillatory frequency rhythms observed in the adults, are not yet fully developed at the age of 12 years (e.g., Krause et al., 2001).

However, due to the observed tendencies, it seems likely that the differences observed in adults, also are of importance for children, if not more so. The specific type of experience may cause the neural changes associated with learning. Thus, handwriting might support the development of these activation patterns in achieving the neural specificity in the brain, including the synchronized theta activity and theta-to-gamma frequency coupling found in the present study. As children continue to improve their language and writing skills throughout adolescence, it is possible that these mechanisms are not yet fully developed at 12 years of age (Krause et al., 2001). Moreover, memory systems involving retrieval might be the last to mature within the brain, suggesting that further research within this field is necessary (Schneider et al., 2016). However, our findings still provide support for handwriting practice providing beneficial neuronal activation patterns for learning. Therefore, maintaining the handwriting skill in school for optimal development seems to be of high importance.

### The Importance of Handwriting Practice in a Learning Environment

Whenever self-generated movements are included as a learning strategy, more of the brain gets stimulated, which results in the formation of more complex neural networks (Van der Meer and Van der Weel, 2017). It also appears that the movements related to keyboard typing do not activate these networks the same way that drawing and handwriting do. Besides, when a child produces individual handwritten letters, the results will be highly variable, leading to a better understanding (Li and James, 2016; James, 2017). The simultaneous spatiotemporal pattern from vision, motor commands, and kinesthetic feedback provided through fine hand movements, is not apparent in typewriting, where only a single button press is required to produce the complete desired form (Longcamp et al., 2006; James, 2010; Vinci-Booher et al., 2016). Therefore, the ongoing replacement of handwriting by keyboard-writing may in some respects seem ill-advised as this appears to negatively affect the learning process (Alonso, 2015; Mangen and Balsvik, 2016). The present findings suggest that the delicate and precisely controlled movements involved in handwriting contribute to the brain’s activation patterns related to learning. We found no evidence of such activation patterns when using a keyboard.

Although it is vital to maintain handwriting practice in school, it is also important to keep up in the continuously developing digital world. Young children should learn to write by hand successfully, and, at the same time learn to manage to write on a keyboard (e.g., learn the touch method and transcribe information fast), depending on the context. The present study shows that the underlying brain electrical activity related to handwriting, typewriting, and drawing is different. Hence, being aware of when to use which strategy is vital, whether it is to learn new conceptual materials or to write long essays. Even though there are underlying differences in the three strategies, it is important to note that the strategies are all cognitive tasks, each serving their own benefits.

## Conclusion

With increasing technological development, it is vital that educators routinely evaluate the influences of learning environments (Stacy and Cain, 2015) for long term implications. It is important to note that the present study did not attempt to suggest that we should prohibit digital devices in the classroom and go back to traditional handwriting in all levels of education. Instead, the purpose was to shed light on the topic and create awareness of which learning tradition has the best effect in what context. When using technological advances, it is important to ensure that handwriting practice remains a central activity in early letter learning, regardless if this occurs with a stylus and tablet or traditional paper and pencil (Vinci-Booher et al., 2016). As digital note-taking has undergone a vast transition, using a digital format today still allows the individual to handwrite notes, add drawings, and highlight text (Stacy and Cain, 2015). Therefore, the benefits from both writing methods can be implemented, and both students and teachers should be conscious of when to use which method. Moreover, learners will also vary in ability, which may affect which learning activities stimulate the use and/or effectiveness of cognitive processes (Arnold et al., 2017).

In conclusion, as Van der Meer and Van der Weel (2017) found evidence for a clear difference in underlying electrical brain activity between typewriting and drawing, this study adds to this knowledge, by showing that typewriting, cursive handwriting, and drawing are each different processes. Nonetheless, handwriting and drawing seem to be more alike compared to typewriting. Therefore, an optimal learning environment needs to include the best from all disciplines, considering the strengths and support each of them offer. This way, both cognitive development and learning efficiency can be strengthened, and pupils and students of all ages and their teachers can keep up with the technological development and digital challenges to come.

## Data Availability Statement

The raw data supporting the conclusions of this article will be made available by the authors, without undue reservation, to any qualified researcher.

## Ethics Statement

The studies involving human participants were reviewed and approved by the Norwegian Regional Ethics Committee (Central Norway). The participants (legal guardian/next of kin) provided written informed consent to participate in this study.

## Author Contributions

EO, FW, and AM contributed equally to the conception, design, analyses and write-up of the work, and were accountable for all aspects of the research. All authors contributed to the article and approved the submitted version.

## Conflict of Interest

The authors declare that the research was conducted in the absence of any commercial or financial relationships that could be construed as a potential conflict of interest.
